# Older Adults as Virtual Subject Matter Experts in COVID Graduate Education

**DOI:** 10.1093/geroni/igab046.2213

**Published:** 2021-12-17

**Authors:** Sonya Barsness

**Affiliations:** Georgetown University, Washington DC, District of Columbia, United States

## Abstract

COVID-19 has further illuminated the need for educational approaches in gerontology that are person-centered and experiential. Ideally, this includes in-person experiences with students and older adults. Through their classroom participation as subject matter experts in aging, older adults share their personal experiences, and react to gerontological theories and ideas. Shared learning offers a platform for exploration of shared humanity, so that older adults are not seen as the “other”, but “us”. This prepares a generation of gerontologists to identify and reject ongoing ageism, again highlighted by the pandemic. COVID-19 has also challenged educators to offer these experiential opportunities. In this presentation we will outline how older adults from a Continuing Care Retirement Community participated virtually in a graduate course. We will discuss how their virtual involvement was structured, how their real-time COVID experiences were integrated, and share feedback from older adult participants and students on their shared learning experiences.

